# Autocrine Fibroblast Growth Factor Receptor 1 Signaling Activates Lactate Dehydrogenase A-Aerobic Glycolysis for Advanced Human Prostate Tumor Growth

**DOI:** 10.1155/proc/8862153

**Published:** 2025-09-16

**Authors:** Xiaoming Xu, Li Wang, Huafeng Pan, Tingitng Gu, Zhongliang Cheng, Tianjun Peng, Jianting Zhang, Jiaren Pan

**Affiliations:** ^1^Department of Urology, Ningbo No. 2 Hospital, Ningbo, Zhejiang, China; ^2^The Sixth People's Hospital of Longgang District, Longgang District, Shenzhen, Guangdong, China; ^3^Loudi Central Hospital, Loudi, Hunan, China

**Keywords:** aerobic glycolysis, autocrine FGF/FGFR, prostate cancer, tumor development

## Abstract

**Background:** Fibroblast growth factor receptor 1 (FGFR1) signaling is activated by fibroblast growth factors (FGFs) during prostate cancer (PCa) progression. However, the mechanisms by which FGFR1 signaling regulates PCa progression are not fully understood. The objective of this study was to investigate the cross talk between autocrine FGF/FGFR1 loop and aerobic glycolysis in progression of advanced PCa.

**Method:** DU145 cells were used as an advanced PCa model. FGFR1 expression was knockdowned by stable expression of anti-FGFR1 shRNA, and lactate dehydrogenase A (LDHA) levels were rescued by ectopic expression of LDHA cDNA. Protein expression was determined using Western blotting and immunohistochemistry. Tumorigenicity of DU145 cells was defined by cell growth, invasion, and survival in both cultures and xenografts in mice.

**Results:** Here, we showed that DU145 cells in cultures expressed both FGF2 and FGFR1, and knockdown of FGFR1 expression or inactivation of FGFR1 signaling reduced LDHA expression or aerobic glycolysis, which was correlated with suppression of both cell proliferation and invasion, and with promotion of apoptosis. Ectopic expression of LDHA cDNA rescued LDHA levels in FGFR1-deficient cells, restoring their aerobic glycolysis, cell growth, and survival. Similarly, the growth rates of xenografted DU145 cells in mice were decreased by the loss of FGFR1 expression but were rescued by the ectopic expression of LDHA.

**Conclusion:** Our data indicate autocrine FGF/FGFR1 signaling regulates aerobic glycolysis in PCa DU145 cells via LDHA, suggesting the potential of targeting FGFs/FGFRs-LDHA for the management of advanced PCa. The regulation of aerobic glycolysis by other growth factors in PCa remains further investigation.

## 1. Introduction

Prostate cancer (PCa) is the most common cancer in men and has been identified as one of diseases causing economic burden in the world [1, 2]. Although some progress has been made in the treatment and diagnosis of PCa, the numbers of new cases and deaths each year are still high, indicated by the fact that there were approximately 1.4 million new cases and 375,000 related death globally in 2020 [3–5]. Therefore, fully understanding signaling pathways that specifically mediate the progression of PCa is urgently needed in an attempt to develop more effective management of PCa.

Fibroblast growth factors (FGFs) belong to a heparin-binding growth factor family with 18 members (FGF1-FGF10, and FGF16-FGF23) that bind to four different signaling tyrosine kinase FGF receptors (FGFRs), FGFR1-4, by either paracrine or autocrine mechanism to exert their functions, including cell proliferation, differentiation, survival, migration, and metabolism [6–9]. There are increased levels of FGF1, FGF2, FGF6, and FGF8 expression in prostate tumor tissues as paracrine and/or autocrine growth factors for tumor growth, and expression of FGFRs, and particularly FGFR1, is most closely linked to PCa progression as well as to castration-resistant PCa (CRPC) bone metastases or to less favorable disease-specific survival in CRPC [10–14]. These findings have been confirmed by experimental studies showing that FGFR1 is one of biomarkers for PCa progression in human prostate LuCaP 23.1 xenograft model [11], and it plays a critical and permissive roles in PCa metastasis in ARR2PB^iPbsn−*Cre*^/TRAMP/*fgfr*1^*loxP*/*loxP*^ transgenic mice [15] as well as in FGFR1-overexpressed human PC3 cell xenograft model [13]. All these studies may indicate the potential of autocrine FGF/FGFR1 signaling as a key regulator for PCa progression and metastasis; however, the molecular details of the FGFR1 signaling remain elusive.

Differing from differentiated cells that use oxidative phosphorylation or anaerobic glycolysis to metabolize glucose, proliferative tumor cells with growth signals metabolize the glucose specifically by aerobic glycolysis (the Warburg effect) to support rapid cell proliferation [16, 17], and the last step of the aerobic glycolysis, from pyruvate to lactate, is catalyzed by lactate dehydrogenase A (LDHA) [18]. Although glucose metabolism in primary or castration-sensitive PCa rarely undergoes a shift toward aerobic glycolysis [19], an enhanced level of the aerobic glycolysis has been found in advanced PCa including neuroendocrine (NE) PCa [20–22] and in patient-derived CRPC xenograft models [23], suggesting that the aerobic glycolysis is at least partially responsible for tumor growth of advanced PCa. How the aerobic glycolysis in prostate tumor cells in advanced PCa is regulated is not fully understood.

Human PCa DU145 cells are an androgen receptor (AR)-negative cells derived from the brain of a Caucasian man (69 years old) with metastatic PCa [24] and have been commonly used as an in vitro model of advanced PCa [25]. Unlike other common PCa cell lines (e.g., PC3 and LNCaP), monolayers of DU145 constitutively secrete FGF2 and express functional FGFR1 protein that can respond to exogenous FGF2 and the heparin-binding fractions of DU145 cell extracts [26–28], indicating that the growth of DU145 cells is at least in part regulated by the autocrine FGF/FGFR1 signaling, a unique model for investigating the role of the autocrine FGFs/FGFR1 in the growth of advanced PCa. The objective of present study was to investigate the cross talk between autocrine FGFs/FGFR1 signaling and aerobic glycolysis in the regulation of advanced PCa DU145 cell growth.

## 2. Materials and Methods

### 2.1. Cell Cultures and Antibodies

The androgen-independent human PCa DU145 cell line was purchased from the cell bank of Shanghai Biology Institute, Chinese Academy of Science (Shanghai, China), and were cultured in Dulbecco's modified Eagle's medium (DMEM) (ThermoFisher Scientific, Waltham, MA, USA) in a 5% CO_2_ incubator at 37°C. The culture medium was supplemented with 10% heat-inactivated fetal bovine serum (FBS, ThermoFisher Scientific) and 1% penicillin–streptomycin (Sigma-Aldrich, St. Louis, MO, USA). For the experiments requiring serum starvation, cells were cultured in DMEM with all the antibiotics, but without FBS supplementation (serum-free medium).

The antibodies used in this study were listed as follows: rabbit anti-human FGFR1 (F5421, Sigma-Aldrich) for Western blot (WB) and immunohistochemical staining (IHC), rabbit anti-human phosphor(p)-FGFR1 (06-1433, Sigma-Aldrich) for WB, rabbit anti-human LDHA (PAS-19754, ThermoFisher Scientific) for WB, mouse anti-β-tubulin (clone KMX-1, MAB3408, Sigma-Aldrich) for WB, and goat anti-rabbit or mouse IgG (secondary antibody) conjugated with a horseradish peroxidase (HRP) (ThermoFisher Scientific).

### 2.2. Generation of Stable shRNA-Expressing DU145 Cell Lines

Both stable FGFR1 shRNA and nontargeting (NT) shRNA DU145 cell lines were generated using the third-generation lentiviral particles as described previously [29]. All three plasmids for lentiviral packaging were received from Addgene (addgene.org): pCMV delta R8.2 (Cat# 12263), pCMV-VSV-G (cat# 8454), and pLKO.1-puro (cat# 8453). A specific shRNA sequence targeting human FGFR1 (FGFR1 shRNA) (5′-CCG GCC ACA GAA TTG GAG GCT ACA ACT CGA GTT GTA GCC TCC AAT TCT GTG GTT TTT-3′) was derived from literature [30], and NT shRNA (5′-CCGGGCGCGATAGCGC TAATAATTTCTCGAGAAATTATTAGCGCTATCGCGCTTTTT-3′) from MISSION® shRNA Plasmid DNA Control Vector SHC016 that does not target any of known genes from any species as described by the manufacturer (Sigma-Aldrich). Both sequences were synthesized by GenScript (Nanjing, Jiangsu, China) and were ligated into pLKO.1-puro backbone to generate the target plasmid pLKO.1-FGFR1-shRNA and pLKO.1-NT-shRNA, respectively. FGFR1 shRNA or NT shRNA lentiviral particles were prepared using HEK293T cells. For virus infection, DU145 cells were infected at 40%–50% confluence with the FGFR1 shRNA and NT shRNA lentiviral particles in polybrene medium to generate FGFR1 shRNA DU145 cells and NT shRNA DU145 cells, respectively. Both types of shRNA-expressing DU145 cells were subsequently incubated in puromycin-containing DMEM (selection medium) for the selection of stably shRNA-expressing cells.

### 2.3. Ectopic Expression of LDHA in FGFR1 shRNA DU145 Cells

Human LDHA cDNA clone (NCBI reference sequence: NM_005566.3) was provided by Sino Biological, Inc. (Shanghai, China), and it was subcloned into pHEX6300 expression vector to create pHEX6300-LDHA for ectopic expression of LDHA. The pHEX6300 vector contains a fused gene (*gfzeo*) encoding zeocin resistance and a green fluorescence protein (GFP) to facilitate both GFP-based cell sorting and antibiotic selection of pHEX6300-expressing cells [31]. NT shRNA and FGFR1 shRNA DU145 cells were transfected with empty pHEX6300 and pHEX6300-LDHA vectors using Lipofectamine 2000 (Invitrogen-GIBCO) and following the manufacturer's protocol to generate NT shRNA-pHEX6300 cells (with empty vector), FGFR1 shRNA-pHEX6300 cells (with empty vector) and FGFR1 shRNA-LDHA cells (with pHEX6300-LDHA), respectively. All these three types of DU145 cells were grown under double antibiotic selections (puromycin and zeocin) and were sorted for GFP enrichment before passaging. GFP expression was stable in subsequent passages, and more than 95% of cells used for experiments showed strong green fluorescence by flow cytometry or microscopy.

### 2.4. Animals and DU145 Xenografts

Male nude BALB/c mice (bodyweight: 0.016–0.021 kg, age: 12–14 weeks old) were obtained from Chinese Academy of Sciences Shanghai Experimental Animal Center (Shanghai, China) and were maintained in microinsulator cages (4 mice per cage) in a pathogen-free insulation facility of the Laboratory Animal Center at Guoke Ningbo Life Science and Health Industry Research Institute (Ningbo, Zhejiang, China), accredited by the Department of Science and Technology of Zhejiang Province in China. In the facility, the animals were housed with a 12-h light–12-h dark cycle at 25 ± 2°C of room temperature and were provided with a standard diet with drinking water *ad libitum*.

DU145 cells were harvested from nonconfluent cultures (70%–80% confluence). After washing with ice-cold PBS, 5 × 10^6^ DU145 cells were suspended in 100 μL Matrigel® Matrix (Corning, NY, USA) and were subcutaneously implanted into the right flank of mice under anesthesia using isoflurane, by which the anesthesia of the mice was induced in a chamber supplied with 2%–3% isoflurane and was subsequently maintained using a nose cone with 1.5% isoflurane during the cell implantation. Mice were randomly divided to three groups to receive different DU145 cells: (1) NT shRNA-pHEX6300 DU145 cells (*n* = 6); (2) FGFR1 shRNA-pHEX6300 DU145 cells (*n* = 8); and (3) FGFR1 shRNA-LDHA DU145 cells (*n* = 8). Both body weight and tumor size were monitored once a week, and the tumor volume was calculated by the formula (mm^3^): tumor volume = length × width^2^/2. After 35 days, tumor tissue from each animal was carefully collected and weighted after euthanasia in a chamber supplied with isoflurane (5%) and subsequently CO_2_. Further analyses of these tissues were performed as appropriate.

All animal procedures were performed under the protocol (No: GK-2021-XM-0009) approved by the Institutional Animal Care and Use Committee of Guoke Ningbo Life Science and Health Industry Research Institute in compliance with National guidelines for the ethical review of laboratory animal welfare (GB/T35892-2018) in China.

### 2.5. Western Blotting

Protein levels in cellular extracts were determined using WBs. Briefly, whole cell lysates were homogenized in lysis buffer (10 mM HEPES [pH 7.9], 10 mM KCl, 0.1 mM EDTA, 0.1 mM EGTA, 0.1% NP 40, 1 mM DTT, and Roche protease inhibitor cocktail), followed by the determination of the protein content using Bio-Rad assay (Bio-Rad Laboratories-Shanghai, Shanghai, China). Protein samples for the Western blotting were prepared by mixing cell lysate with an equal volume of 2× SDS sample buffer (20 mM Tris–HCl [pH 6.8], 5% [wt/vol] SDS, 10% [vol/vol] mercaptoethanol, 2 mM EDTA, and 0.02% bromophenol blue) and were boiled for 5 min. Approximately 100 μg of total protein from each sample was fractionated by SDS-PAGE and transferred to nitrocellular membranes (Bio-Rad Laboratories), blocked with 5% fat-free milk in TBS-T (20 mM Tris–HCl, pH 7.6, 137 mM NaCl, 0.1% Tween 20) for 1 h, and then probed with the appropriate antibodies as mentioned above in TBS containing 2.5% of milk at 4°C overnight. The specific protein binds recognized by the antibodies on the membrane were visualized using Amersham ECL Detection Reagents (Cytiva, Shanghai, China). Blots were reprobed using antitubulin monoclonal IgG to confirm equal protein loading of each sample if possible.

### 2.6. Determination of Lactate Levels Using the Hydrazine-Sink Method

L-lactate in DU145 cell cultures was measured using lactate dehydrogenase (LDH) and the hydrazine-sink method. In brief, after overnight incubation, supernatant was collected from cell cultures for lactate measurement using the hydrazine-sink method as described previously [32]. In brief, the supernatant and lactate standards were incubated in 96-well plates in the presence of NAD^+^ (2 mM) and LDH (1 U/mL) in a glycine–hydrazine buffer (pH 9.2). The lactate was oxidized by LDH to produce equimolar NADH, detectable at 340 nm. Lactate production rates were expressed relative to cell numbers in cultures.

### 2.7. Enzyme-Linked Immunosorbent Assay (ELISA) of FGF2

DU145 cells were plated at 1 × 10^4^ cells/well in 24-well plates in DMEM containing 10% FBS for 24 h and then were washed with serum-free medium, followed by an additional 48 h of culture with serum-free medium. The culture supernatant was collected and centrifuged, and the levels of FGF2 in the supernatants were determined using human FGF basic ELISA Quantikine assay kit (Cat#: DFB50, R&D Systems, Inc., Minneapolis, MN, USA) according to the manufacturer's instruction. Data were expressed as an average of measurements performed in triplicate relative to cell numbers in cultures for each sample.

### 2.8. Determination of Cell Invasion

Cell invasion was determined using Transwell assay as described previously [33]. In brief, DU145 cells (1 × 10^3^ cells/well) were seeded into the Matrigel-coated upper chamber with serum-free medium. The lower chamber was added with 500 μL of medium containing 10% FBS. After incubation for 24 h at 37°C to allow invasion of the cells through the membrane, the nonmigrated cells were removed with cotton swabs. Then, cells were stained with 0.1% crystal violet for 15 min, and invasion cells in randomly selected five visual fields were counted under a light microscope in a blinded fashion.

### 2.9. Cell Growth

DU145 cell growth in cultures was determined using CyQUANT XTT assay (ThermoFisher Scientific). Briefly, cells were seeded in 96-well plates at a density of 2 × 10^3^ cells/well/0.1 mL and were incubated in 5% CO_2_ atmosphere at 37°C. After 0, 1, 2, and 3 days of incubation, 70 μL of XTT solution, prepared by mixing 1 mL of electron coupling reagent with 6 mL of XTT, was added to each well for 4 h according to manufacturer's protocol. The optical density (OD) was read at both 450 and 660 nm using a plate reading spectrophotometer. The OD value, representing viable cell density, was reported as the absorbance of the sample at 450 nm minus the absorbance of the sample at 660 nm minus the absorbance of the blank at 450 nm. Finally, the cell growth (increase in the cell density) at those different time points was calculated.

### 2.10. Cell Apoptosis

Cell apoptosis or survival of different DU145 cell cultures under serum deprivation with 250 μM H_2_O_2_ was measured by using FACS analysis with 7-aminoactinomycin D (7-AAD) and Annexin-V conjugated with fluorescein (FITC) or phycoerythrin (PE) following the manufacturer's protocol (ThermoFisher Scientific). Annexin-V FITC/PE was used for detection of early apoptosis and 7-AAD for late apoptosis staining. Briefly, monolayers of DU145 cells were released by a brief incubation with trypsin–EDTA solution and then incubated with Annexin-V-FITC/PE and 7-AAD for 15 min at room temperature in the dark. The intensity of fluorescence of apoptotic cells was measured by a flow cytometry and analyzed compared with background controls using FlowJo software (Tree Star, Inc., Ashland, OR). Thus, in a typical FACS graph, nonapoptotic (viable) cells were in the lower left quadrant (Q3), necrotic cells in the upper left quadrant (7-AAD-positive only) (Q1), late apoptotic cells in the upper right quadrant (both Annexin-V and 7-AAD-positive) (Q2), and early apoptotic cells in the lower right quadrant (Annexin-V-positive only) (Q4).

### 2.11. Measurement of Extracellular Acidification Rate (ECAR)

ECAR in different DU145 cells was determined by using Agilent Seahorse XF Glycolysis Stress Test kits following manufacturer's instruction (Agilent Technologies, Santa Clara, CA, USA). In brief, DU145 cells (1 × 10^4^ cells/well) in DMEM supplemented with 10% FBS were cultured in 96-well XF Seahorse microplates in a CO_2_ incubator overnight after 1 h of rest at room temperature, followed by changing the DMEM to warmed assay medium (2 mM glutamine containing Seahorse XF base medium). After 1 h of rest in a non-CO_2_ incubator at 37°C, the cell culture microplate along with the glucose/oligomycin/2-deoxy-glucose (2-DG)-loaded sensor cartridge was loaded into the Seahorse XFe/XF96 Analyzer. The ECAR assay was run by sequential injection of 25 mM glucose, 1 M oligomycin, and 100 mM 2-DG into each well at the specified time point for measurement of baseline (0–15 min), glycolysis (with glucose, 15–35 min), glycolytic capacity (with oligomycin, 35–55 min), and glycolytic reserve (with 2-DG, from 55 to 70 min), respectively. The data were analyzed by using Agilent Seahorse Wave software, and ECAR was reported in mpH/min.

### 2.12. IHC of FGFR1

Tumor tissues from nude BALB/c mice were immediately fixed in formalin, followed by paraffin-embedded. Tissue sections were cut at 4 μm thickness. Immunostaining of FGFR1 in the tissue sections was performed using an Extravidin–biotin complex (ABC) method as outlined by the manufacturer (Sigma-Aldrich). Primary anti-FGFR1 antibody (Cat# F5421, Sigma-Aldrich) was diluted at a predetermined optimal ratio of 1:100 (anti-FGFR1) in 0.3% BSA-containing PBS. After overnight incubation with the anti-FGFR1 antibody at 4°C, the tissue sections were rinsed in PBS and incubated for 1 h at room temperature with a biotinylated goat anti-rabbit antibody. Endogenous peroxidase was quenched by incubation in 0.3% H_2_O_2_ in methanol for 30 min; and the tissue sections were rinsed in PBS and incubated for 30 min with the ABC solution. After further washes in PBS, the reaction product was visualized using diaminobenzidine (Sigma-Aldrich), and the sections were counterstained with Harris's hematoxylin, dehydrated in graded alcohols, cleared in xylene, and mounted. The FGFR1 expression was represented as mean integrated OD or staining intensity of randomly selected at least 20 microscopic fields per sample, and it was determined using Image-Pro plus 6.0 software (media cybernetics, Rockville, MD, USA).

### 2.13. Determination of Intratumoral LDHA Using ELISA

The tumor tissue was minced into small pieces and rinsed with ice-cold PBS to remove the remaining blood, followed by homogenization in PBS at a ratio of 1 g of tissue weight to 9 mL of PBS with a glass homogenizer on ice. The homogenate was further sonicated, and tissue supernatant was collected by centrifugation at 5000 ×g for 10 min. The amount of LDHA in the tissue supernatants along with LDHA standards was determined using human LDHA ELISA kit (Cat# D711116) following manufacturer's protocol (Sangon Biotech, Shanghai, China). The content of LDHA protein in the tissue supernatant was expressed relative to the amount of its total protein.

### 2.14. Statistical Analysis

All data were presented as the mean ± standard deviation (SD). The difference between groups was analyzed using two-tailed *t*-test or analysis of variance (ANOVA) for the comparisons using GraphPad Prism 9 (GraphPad Software, Inc., San Diego, CA, USA). Less than 0.05 (< 0.05) of *p* value was considered significant.

## 3. Results

### 3.1. FGFR1 Signaling is an Autocrine Growth Regulator of DU145 Cells in Cultures

**Figure 1:**
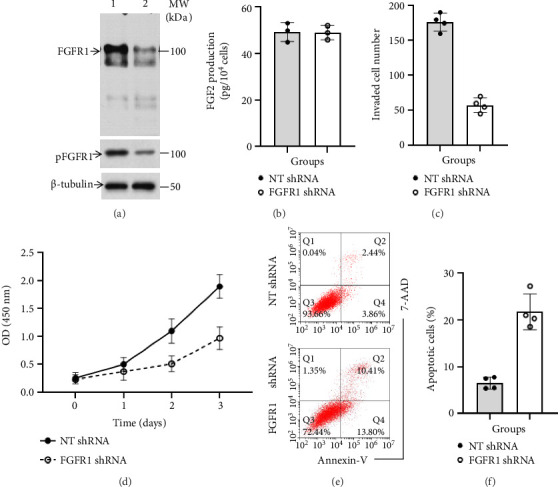
Effect of knockdown of FGFR1 expression on cell proliferation, invasion, and survival in cultured DU145 cells. (a) Protein levels of both FGFR1 and pFGFR1 along with loading control β-tubulin in whole cell lysate of NT shRNA DU145 cells (lane 1) versus FGFR1 shRNA DU145 cells (lane 2). Data were a representative of three separate experiments. (b) FGF2 release in NT shRNA DU145 cell cultures versus FGFR1 shRNA DU145 cell cultures. Data were presented as mean ± standard deviation (SD) of three separate experiments, *p*=0.9142 (two-tailed *t*-test, *n* = 3). (c) Cell migration of NT shRNA DU145 cells versus FGFR1 shRNA DU145 cells in cultures. Data were presented as mean ± SD of four separate experiments, *p* < 0.0001 (two-tailed *t*-test, *n* = 4). (d) Proliferation of NT shRNA DU145 cells versus FGFR1 shRNA DU145 cells in 10%FBS-containing DMEM. Data were presented as mean ± SD of three separate experiments, *p* < 0.0001 (two-way ANOVA, *n* = 3). (e) A typical FACS graph displaying cell death of NT shRNA DU145 cells and FGFR1 shRNA DU145 cells after exposure to H_2_O_2_. (f) The sum of early (Q4) and late apoptotic cells (Q3) in NT shRNA DU145 cells versus FGFR1 shRNA DU145 cells. Data were presented as mean ± SD of four separate experiments, *p*=0.0003 (two-tailed *t*-test, *n* = 4).

WB analysis showed that stable expression of FGFR1 shRNA resulted in knockdown of both total and phosphorylated FGFR1 protein levels (Figure 1(a)), but not significantly affected secretion of FGF2 in cultured DU145 cells (Figure 1(b)). Further analyses indicated that as compared with NT shRNA-expressing DU145 cells, DU145 cells with reduced active pFGFR1 exhibited the lower rates of cell migration (NT shRNA: 176.25 ± 12.5; FGFR1 shRNA: 57.5 ± 10.41, *p* < 0.0001, two-tailed *t*-test, *n* = 4) (Figure 1(c)) and cell growth (TN shRNA vs. FGFR1 shRNA, *p* < 0.0001, two-way ANOVA, *n* = 3) (Figure 1(d)), which were associated with an increase in cell apoptosis (NT shRNA: 6.55 ± 1.28%; FGFR1 shRNA: 21.8 ± 3.8, *p*=0.0003, two-tailed *t*-test, *n* = 4). Taken together, these data suggested that the FGF2/FGFR1 loop was an important autocrine growth regulator of PCa DU145 cells.

### 3.2. FGFR1 Signaling Regulates Aerobic Glycolysis in DU145 Cells in Cultures

**Figure 2:**
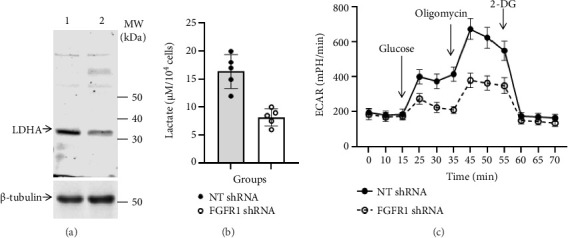
Effect of knockdown of FGFR1 expression on LDHA expression and activity in cultured DU145 cells. (a) Protein levels of LDHA in whole cell lysate of NT shRNA DU145 cells (Lane 1) versus FGFR1 shRNA DU145 cells (Lane 2). The molecular weight of LDHA is approximately 37 kDa. Data were a representative of three separate experiments. (b) Lactate production in NT shRNA DU145 cell cultures versus FGFR1 shRNA DU145 cell cultures. Data were presented as mean ± SD of five separate experiments, *p*=0.007 (two-tailed *t*-test, *n* = 5). (c) Aerobic glycolysis of NT shRNA DU145 cell cultures versus FGFR1 shRNA DU145 cell cultures. Data were presented as mean ± SD of three separate experiments, *p* < 0.0001 in both glycolysis and glycolytic capacity (two-way ANOVA, *n* = 3).

The aerobic glycolysis is the metabolism of glucose to lactate in the presence of oxygen, which is an essential part of metabolic reprogramming for cancer progression [34]. To investigate whether the aerobic glycolysis is regulated by FGFR1 signaling in DU145 cells, the effect of FGFR1 inactivation on LDHA expression and its activities was investigated. As shown in Figure 2(a), knockdown of FGFR1 expression or inactivation of its signaling resulted in a decrease in expression of LDHA protein in DU145 cells, which was consistent with a decrease in lactate production (8.22 ± 1.51 μM/10^4^ cells) in FGFR1 shRNA cell cultures as compared to 16.4 ± 3.05 μM/10^4^ cells in NT shRNR cell cultures (*p*=0.0007, two-tailed *t*-test, *n* = 5) (Figure 2(b)). The difference in aerobic glycolysis between NT shRNA and FGFR1 shRNA DU143 cells was further measured by the ECAR assay. As shown in Figure 2(c), upon the addition of glucose (glycolysis), ECAR was significantly stimulated from basal levels, 185 ± 30 mPH/min to 400 ± 40 mPH/min in NT shRNA cells versus 175 ± 20 mPH/min to 275 ± 30 mPH/min in FGFR1 shRNA cells (approximately 2.15-fold loss of glycolysis, NT shRNA vs. FGFR1 shRNA, *p* < 0.0001, two-way ANOVA, *n* = 3). Addition of oligomycin (glycolytic capacity) that inhibited oxidative phosphorylation further increased ECAR to 675 ± 60 mPH/min in NT shRNA cells versus 380 ± 40 mPH/min in FGFR1 shRNA cells (approximately 2.39-fold loss of glycolytic capacity, NT shRNA vs. FGFR1 shRNA, *p* < 0.0001, two-way ANOVA, *n* = 3). Taken together, these results suggested that FGFR1 signaling positively regulated glycolysis via LDHA activity in DU145 cells.

### 3.3. Ectopic Expression of LDHA Rescues Aerobic Glycolysis in FGFR1-Deficient DU145 Cells in Cultures

**Figure 3:**
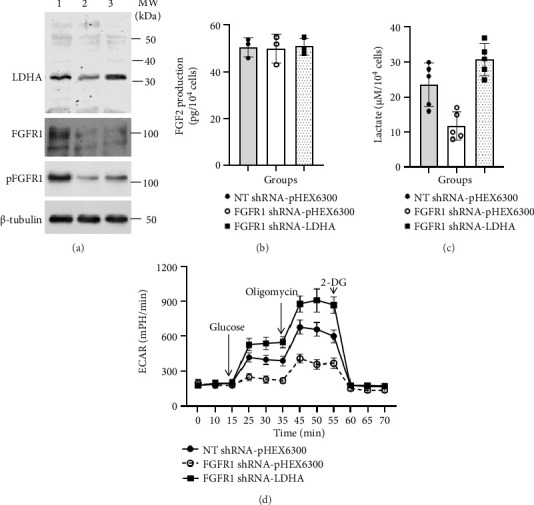
Effect of ectopic expression of LDHA on aerobic glycolysis in FGFR1 shRNA DU145 cells. (a) Protein levels of LDHA, FGFR1, and pFGFR1 along with loading control β-tubulin in whole cell lysate of NT shRNA-pHEX6300 DU145 cells (Lane 1), FGFR1 shRNA-pHEX6300 DU145 cells (Lane 2), and FGFR1 shRNA-LDHA DU145 cells (Lane 3). Data were a representative of three separate experiments. (b) FGF2 release in NT shRNA-pHEX6300 DU145 cell cultures, FGFR1 shRNA-pHEX6300 DU145 cell cultures, and FGFR1 shRNA-LDHA DU145 cell cultures. Data were presented as mean ± SD of three separate experiments, *p*=0.9671 (one-way ANOVA, *n* = 3). (c) Lactate production in NT shRNA-pHEX6300 DU145 cell cultures, FGFR1 shRNA-pHEX6300 DU145 cell cultures, and FGFR1 shRNA-LDHA DU145 cell cultures. Data were presented as mean ± SD of five separate experiments, NT shRNA-pHEX6300 versus FGFR1 shRNA-pHEX6300: *p*=0.0005 (two-tailed *t*-test, *n* = 5), NT shRNA-pHEX6300 versus FGFR1 shRNA-LDHA: *p*=0.01442 (two-tailed *t*-test, *n* = 5), and FGFR1 shRNA-pHEX6300 versus FGFR1 shRNA-LDHA: *p* < 0.0001 (two-tailed *t*-test, *n* = 5). (d) Aerobic glycolysis of NT shRNA-pHEX6300 DU145 cell cultures, FGFR1 shRNA-pHEX6300 DU145 cell cultures, and FGFR1 shRNA-LDHA DU145 cell cultures. Data were presented as mean ± SD of three separate experiments, NT shRNA-pHEX6300 versus FGFR1 shRNA-pHEX6300 in both glycolysis and glycolytic capacity: *p* < 0.0001 (two-way ANOVA *n* = 3), NT shRNA-pHEX6300 versus FGFR1 shRNA-LDHA in both glycolysis and glycolytic capacity: *p* < 0.0001 (two-way ANOVA *n* = 3), and FGFR1 shRNA-pHEX6300 versus FGFR1 shRNA-LDHA: *p* < 0.0001 (two-tailed *t*-test, *n* = 3).

LDHA was ectopically expressed in FGFR1 shRNA (LDHA-deficient) DU145 cells by stable expression of human LDHA cDNA using pHEX6300 vector (FGFR1 shRNA-LDHA), whereas NT shRNA and FGFR1 shRNA DU145 cells with stable expression of empty pHEX6300 vector were used as control cells, NT shRNA-pHEX6300 and FGFR1 shRNA-pHEX6300 DU145 cells, respectively. WB analysis indicated that the LDHA protein level was significantly increased by stable expression of LDHA cDNA in FGFR1 shRNA-LDHA cells, but FGFR1 protein or signaling remained deficient as compared to that in FGFR1 shRNA-pHEX6300 cells (Figure 3(a)). Again, the increased LDHA protein in FGFR1 shRNA-LDHA cells was positively correlated with more production of lactate (25.4 ± 3.34 μM/10^4^ cells vs. 8.2 ± 2.59 μM/10^4^ cells in FGFR1 shRNA-pHEX6300 cells, *p* < 0.0001, two-tailed *t*-test, *n* = 5) (Figure 3(c)) as well as with higher ECAR values after addition of both glucose (glycolysis) and oligomycin (glycolytic capacity) in FGFR1 shRNA-LDHA cells as compared to those of FGFR1 shRNA-pHEX6300 cells (*p* < 0.0001, two-way ANOVA, *n* = 3) (Figure 3(d)). It was worth paying attention to that a close correlation of LDHA with aerobic glycolysis was also seen between NT shRNA-pHEX6300 cells and FGFR1 shRNA-LDHA cells even though the FGFR1 signaling was normal in NT shRNA-pHEX6300 but deficient in FGFR1 shRNA-LDHA cells (Figure 3(a)), in which the higher LDHA protein level in FGFR1 shRNA-LDHA was correlated with higher levels of lactate production (FGFR1 shRNA-LDHA vs. NT shRNA-pHEX6300, *p*=0.0144, two-tailed *t*-test, *n* = 5) and with higher ECAR values in both glycolysis (with glucose) and glycolytic capacity (with oligomycin) (FGFR1 shRNA-LDHA vs. NT shRNA-pHEX6300, *p* < 0.0001, two-way ANOVA, *n* = 3) in these cells. These data might suggest that LDHA was a key determinant of FGFR1 signaling-dependent aerobic glycolysis in DU145 cells.

**Figure 4:**
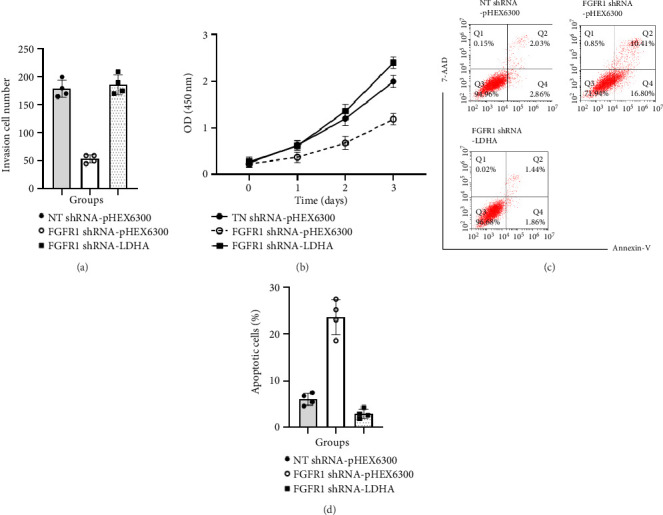
Effect of ectopic expression of LDHA on cell proliferation, migration, and survival in FGFR1 shRNA DU145 cells. (a) Cell migration of NT shRNA-pHEX6300 DU145 cells, FGFR1 shRNA-pHEX6300 DU145 cells, and FGFR1 shRNA-LDHA DU145 cells in cultures. Data were presented as mean ± SD of four separate experiments, NT shRNA-pHEX6300 versus FGFR1 shRNA-pHEX6300: *p* < 0.0001 (two-tailed *t*-test, *n* = 4), NT shRNA-pHEX6300 versus FGFR1 shRNA-LDHA: *p*=0.5504 (two-tailed *t*-test, *n* = 4), and FGFR1 shRNA-pHEX6300 versus FGFR1 shRNA-LDHA: *p* < 0.0001 (two-tailed *t*-test, *n* = 4). (b) Proliferation of NT shRNA-pHEX6300 DU145 cells, FGFR1 shRNA-pHEX6300 DU145 cells, and FGFR1 shRNA-LDHA DU145 cells in 10%FBS-containing DMEM. Data were presented as mean ± SD of three separate experiments, NT shRNA-pHEX6300 versus FGFR1 shRNA-pHEX6300: *p* < 0.0001 (two-way ANOVA, *n* = 3), NT shRNA-pHEX6300 versus FGFR1 shRNA-LDHA: *p*=0.0099 (two-way ANOVA, *n* = 3), and FGFR1 shRNA-pHEX6300 versus FGFR1 shRNA-LDHA: *p* < 0.0001 (two-way ANOVA, *n* = 3). (c) A typical FACS graph displaying cell death of NT shRNA-pHEX6300 DU145 cells, FGFR1 shRNA-pHEX6300 DU145 cells, and FGFR1 shRNA-LDHA DU145 cells after exposure to H_2_O_2_. (d) The sum of early (Q4) and late apoptotic cells (Q2) in NT shRNA-pHEX6300 DU145 cells, FGFR1 shRNA-pHEX6300 DU145 cells, and FGFR1 shRNA-LDHA DU145 cells. Data were presented as mean ± SD of four separate experiments, NT shRNA-pHEX6300 versus FGFR1 shRNA-pHEX6300: *p*=0.0001 (two-tailed *t*-test, *n* = 4), NT shRNA-pHEX6300 versus FGFR1 shRNA-LDHA: *p*=0.0092 (two-tailed *t*-test, *n* = 4), and FGFR1 shRNA-pHEX6300 versus FGFR1 shRNA-LDHA: *p* < 0.0001 (two-tailed *t*-test, *n* = 4).

To further confirm whether the rescued aerobic glycolysis by ectopic expression of LDHA could result in the growth recovery of FGFR1 shRNA DU145 cells, the cell migration, proliferation, and survival of FGFR1 shRNA-LDHA were examined as compared with those of FGFR1 shRNA-pHEX6300. As shown in Figure 4, the number of migrated cells in FGFR1 shRNA-LDHA (186.25 ± 17.97) was significantly higher than that (53.75 ± 7.5) in FGFR1 shRNA-pHEX6300 (*p* < 0.0001, two-tailed *t*-test, *n* = 4) and was not different from that (178.75 ± 15.48) in NT shRNA-pHEX6300 (*p*=0.5504, two-tailed *t*-test, *n* = 4) (Figure 4(a)). The cell growth of FGFR1 shRNA-LDHA was significantly recovered as compared with that of FGFR1 shRNA-pHEX6300 (*p* < 0.0001, two-way ANOVA, *n* = 3) and of NT shRNA-pHEX6300 (*p*=0.0099, two-way ANOVA, *n* = 3) (Figure 4(b)). Further, the rescued aerobic glycolysis contributed to less cell death, indicated by that the apoptosis (2.9 ± 1.04%) in FGFR1 shRNA-LDHA was significantly lower than that (23.63 ± 3.81%) in FGFR1 shRNA-pHEX6300 (*p* < 0.0001, two-tailed *t*-test, *n* = 4) and (6.08 ± 1.32%) in NT shRNA-pHEX6300 (*p*=0.0092, two-tailed *t*-test, *n* = 4) (Figure 4(c)).

### 3.4. FGFR1/LDHA-Regulated Aerobic Glycolysis Enhances the Growth of Xenografted DU145 Cells in Mice

**Figure 5:**
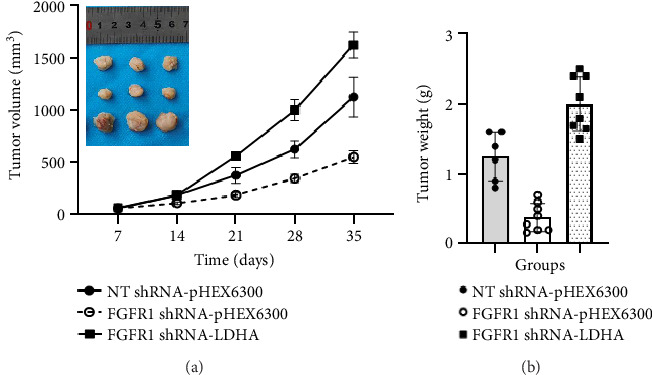
Effects of both FGFR1 knockdown and ectopic LDHA expression on tumor growth of xenografted DU145 cells in mice. The NT shRNA-pHEX6300 DU145 cells (*n* = 6), FGFR1 shRNA-pHEX6300 DU145 cells (*n* = 8), and FGFR1 shRNA-LDHA DU145 cells (*n* = 8) were xenografted in nude BALB/c mice. (a) Tumor growth rates of each group were determined by measurement of tumor volume at each time point. Data were presented as mean ± SD of 6–8 tumors/mice. NT shRNA-pHEX6300 versus FGFR1 shRNA-pHEX6300: *p* < 0.0001 (two-way ANOVA), NT shRNA-pHEX6300 versus FGFR1 shRNA-LDHA: *p* < 0.0001 (two-way ANOVA), and FGFR1 shRNA-pHEX6300 versus FGFR1 shRNA-LDHA: *p* < 0.0001 (two-way ANOVA). Insert image: 3 typical tumor tissues from each group at the experimental endpoint (on Day 35). (b) Tumor weight of each group at the experimental endpoint (on Day 35). Data were presented as mean ± SD of 6–8 tumors/mice. NT shRNA-pHEX6300 versus FGFR1 shRNA-pHEX6300: *p* < 0.0001 (two-tailed *t*-test), NT shRNA-pHEX6300 versus FGFR1 shRNA-LDHA: *p*=0.0028 (two-tailed *t*-test), and FGFR1 shRNA-pHEX6300 versus FGFR1 shRNA-LDHA: *p* < 0.0001 (two-tailed *t*-test).

To further investigate the role of LDHA in FGFR1-regulated prostate tumor growth, the growth of xenografted DU145 with different expression of FGFR1 (NT shRNA vs. FGFR1 shRNA) or LDHA (FGFR1 shRNA-pHEX6300 vs. FGFR1 shRNA-LDHA) was investigated. As shown in Figure 5, the growth rate (tumor volume change per a given time period) of NT shRNA-pHEX6300 (*n* = 6) was significantly faster than that of FGFR1 shRNA-pHEX6300 (*n* = 8) (*p* < 0.0001, two-way ANOVA) (Figure 5(a)), and the similar result was seen when the rate of FGFR1 shRNA-LDHA was compared with that of FGFR1 shRNA-pHEX6300 (*p* < 0.0001, two-way ANOVA, *n* = 8) (Figure 5(a)). These data were confirmed by the tumor weight at the experimental endpoint in each group, indicated by the fact the weight of tumor tissue (1.25 ± 0.35 g) in NT shRNA-pHEX6300 was significantly heavier than that (0.38 ± 0.2 g) in FGFR1 shRNA-pHEX6300 (*p* < 0.0001, two-tailed *t*-test) that was significantly increased by ectopic expression of LDHA to 2.01 ± 0.39 g in FGFR1 shRNA-LDHA (*p* < 0.0001, two-tailed *t*-test) (Figure 5(b)).

**Figure 6:**
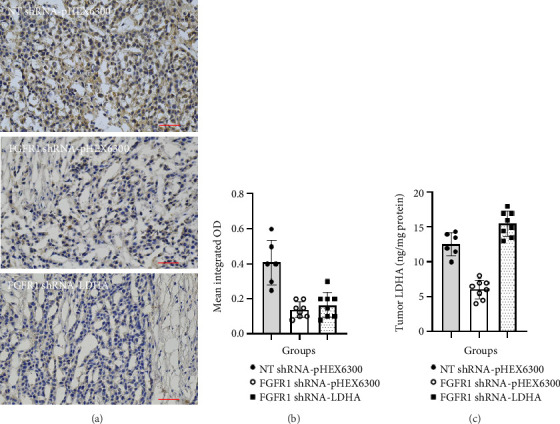
Expression of both FGFR1 and LDHA in xenografted DU145 cells in mice. (a) A typical microscopic view of immunochemical staining of human FGFR1 in tissue section of each group at the experimental endpoint (on Day 35). Bar: 20 μm. (b) The levels of FGFR1 expression were represented as mean integrated OD or FGFR1 staining intensity in tissue sections of NT shRNA-pHEX6300 (*n* = 6), FGFR1 shRNA-pHEX6300 (*n* = 8), and FGFR1 shRNA-LDHA (*n* = 8). Two nonserial sections of each tumor/mouse were used for FGFR1 staining. NT shRNA-pHEX6300 versus FGFR1 shRNA-pHEX6300: *p* < 0.0001 (two-tailed *t*-test), NT shRNA-pHEX6300 versus FGFR1 shRNA-LDHA: *p*=0.0004 (two-tailed *t*-test), and FGFR1 shRNA-pHEX6300 versus FGFR1 shRNA-LDHA: *p*=0.3725 (two-tailed *t*-test). (c) The intratumoral LDHA levels of tumor specimens from NT shRNA-pHEX6300 (*n* = 6), FGFR1 shRNA-pHEX6300 (*n* = 8), and FGFR1 shRNA-LDHA (*n* = 8) were determined using human ELISA kit. NT shRNA-pHEX6300 versus FGFR1 shRNA-pHEX6300: *p* < 0.0001 (two-tailed *t*-test), NT shRNA-pHEX6300 versus FGFR1 shRNA-LDHA: *p*=0.0328 (two-tailed *t*-test), and FGFR1 shRNA-pHEX6300 versus FGFR1 shRNA-LDHA: *p* < 0.0001 (two-tailed *t*-test).

To verify the role of FGFR1/LDHA in the growth of xenografted DU145 cells, the expression of both FGFR1 and LDHA was confirmed in the tissues of these experimental groups (Figure 6(a)). Similar to the results in DU145 cell cultures, the IHC staining intensity of FGFR1 protein in NT shRNA-pHEX6300 (OD score: 0.42 ± 0.12, *n* = 6) was significantly higher than that in FGFR1 shRNA-pHEX6300 (OD score: 0.14 ± 0.05, *n* = 8) (*p* < 0.0001, two-tailed *t*-test) as well as in FGFR1 shRNA-LDHA (OD score: 0.17 ± 0.07, *n* = 8) (*p*=0.0003, two-tailed *t*-test) (Figure 6(b)). The intratumor LDHA was decreased in FGFR1 shRNA-pHEX6300 (5.88 ± 1.56 ng/mg, *n* = 8) as compared with that (12.0 ± 2.28 ng/mg, *n* = 6) in NT shRNA-pHEX6300 (*p* < 0.0001, two-tailed *t*-test), and it was rescued or elevated in FGFR1 shRNA-LDHA (14.75 ± 1.98 ng/mg, *n* = 8) (FGFR1 shRNA-pHEX6300 vs. FGFR1 shRNA-LDHA, *p* < 0.0001, two-tailed *t*-test) (Figure 6(c)). Taken together, these data suggested that the intratumor LDHA expression that might be regulated by autocrine FGFR1 signaling played an important role in the xenograft growth of DU145 cells in mice.

## 4. Discussion

It has been long recognized that tumor cells are frequently found to have an ability to regulate their own growth by release of autocrine growth modulatory substances, which are the most important for tumor growth and progression [35, 36]. Evidence in literature has shown that dysregulated FGF/FGFR signaling is associated with aggressive cancer phenotypes [37]. Indeed, in prostate tumor tissues, upregulated expression of FGFs including FGF2 as paracrine/or autocrine growth factors for PCa cells, and their receptor FGFRs such as FGFR1 are positively correlated with PCa progression [12, 38, 39], suggesting that FGFs/FGFRs systems might be an important autocrine regulator for PCa cell growth. However, the signaling pathways by which the FGFs/FGFRs system as an autocrine regulator for PCa progression are not well defined. Human prostate DU145 cell line is a representative cell line of metastatic or advanced PCa [24, 25], and it expresses FGF2-FGFR1 system [26–28]. In addition to FGF2, DU145 cells have been found to express FGF8 mRNA [40] and FGF17 (mRNA and protein) [41], suggesting that FGF8/FGF17 may also activate FGFR1 in this PCa model. The present study has shown that knockdown of FGFR1 expression in DU145 cells results in reducing cell proliferation, migration, and survival from H_2_O_2_-induced cell death, which is associated with reduction of LDHA expression, lactate production, and extracellular acidification, the measures of aerobic glycolysis in tumor cells [18]. These data may suggest that the autocrine FGFs-FGFR1 regulation of DU145 PCa cell growth is mediated by LDHA-dependent aerobic glycolysis. The role of LDHA-aerobic glycolysis in the autocrine FGFs-FGFR1 regulation is further confirmed by the experiments with rescued expression of LDHA in FGFR1-deficient DU145 cells, showing that the rescued LDHA expression in FGFR1-deficient DU145 cells could promote the cell proliferation, migration, and survival from oxidative stress-induced cell death in cultures as well as tumorigenicity of xenografted DU145 PCa in mice.

It has been well documented that abnormal proliferation of tumor cells is induced by the mutations of proto-oncogenes and tumor-suppressive genes in coordination with autocrine growth factors or their receptors [35] and tumor-specific reprogramming of energy metabolism-aerobic glycolysis [17]. An increase in aerobic glycolysis has also been confirmed in advanced PCa including NEPCa subtypes [20–22], in which approximately 85% pyruvate is conversed to lactate by LDHA to complete the aerobic glycolytic pathway [42], leading to excessive accumulation of lactic acid as seen increased ECAR. Taken together, the autocrine FGFs/FGFRs loop may regulate the aerobic glycolysis for the progression of advanced PCa or different CRPC subtypes.

Autocrine signaling of a secreted factor binding to its receptor can contribute to four different (patho)physiological roles [43]: negative feedback loops, positive feed-forward loops, self-stimulation, and receptor signaling cross talk or transactivation. Our data show that the production of the secreted FGF2, measured by ELISA, was not affected by knockdown or inactivation of FGFR1, suggesting that the FGF2/FGFR1 autocrine loop may be neither negative feedback nor positive feed-forward or self-stimulation via any of FGFR1 intracellular signaling pathways. Instead, there may be a more elaborate “transactivation” process between FGF2 secretion and FGFR1 activation in DU145 cells. Indeed, the transcription of FGF2 mRNA results in producing low-molecular weight FGF2 (LMW FGF2) as well as high-molecular weight FGF2 (HMW FGF2) [44], and they function in different mechanisms, by which LMW FGF2 activates FGFR either by interaction with cell surface heparan sulfate proteoglycans and FGFR on the cell surface or by endocytosis of activated FGF-FGFR complexes in the cytosol and nucleus of the cells, while the HMW FGF2 localized to the nucleus and signals independently of FGFRs [37]. This may explain at least one reason why the FGFs/FGFR1 autocrine loop did not affect the subsequent release of LMW FGF2. However, the pathways by which FGFs/FGFR1 signaling interacts with the aerobic glycolysis in advanced PCa are barely understood.

An early study has shown that activation of FGFR1 can directly phosphorylate LDHA that promotes the Warburg effect and tumor growth by regulating NADH/NAD^+^ redox homeostasis in human tumor cells [45], and deletion of FGFRs expression does not affect *LDHA* transcript levels but promotes the degradation of LDHA protein in DU145 cells [46]. In present study, we also found that reduction of FGFR1 or pFGFR1 expression resulted in decreasing LDHA protein level. The other lines of evidence support the regulation of LDHA by FGFRs in activation of aerobic glycolysis of advanced PCa are as follows: (1) in parallel with upregulated expression of FGFs and their receptor FGFRs [12, 38], LDHA expression is higher in prostate tumors than those in prostate nontumor tissues [47–49], and it tends to increase with increasing Gleason score [49], (2) both FGFR1 knockdown in present study and LDHA knockdown in a published study [48] in DU145 cells show similar results, evidenced by the significant increase of cell apoptosis and reduction of cell migration, invasion and glycolysis, and (3) ectopic expression of LDHA in FGFR1-deficient DU145 cell could rescue cell proliferation, migration, and aerobic glycolysis in cultures and tumor growth in a xenograft model. Taken together, these data may suggest that upon activation by FGFs (i.e., FGF2, FGF8, and/or FGF17), FGFR1 signaling interacts with activation of LDHA-aerobic glycolysis of AR-independent DU145 cells, which, however, remains further investigation.

Prostate tumor initially requires androgen for growth, and it responds to treatment with androgen deprivation therapy and AR-targeted therapy [50, 51]. After this primary treatment, prostate tumors in patients progress to a state of androgen independence or CRPC [50]; thus, fully understanding molecular mechanisms by which CRPC cell growth is urgently needed for development of more specific and effective therapies in the management of patients with CRPC. Analysis of molecular landscapes suggests that metastatic CRPC can be divided to several subtypes including AR-positive PCa (ARPC), AR-low PCa (ARLPC), and double (AR and NE)-negative PCs (DNPC) [52, 53], and the levels of *FGFs* and *FGFRs* transcripts are expressed at different frequencies but are significantly higher in AR-null DNPC than those in ARPC tissues, which is correlated with elevated mitogen-activated protein kinase kinase (MEK) or FGFR pathway activation in DNPC as well as ARLPC [52, 53], suggesting that the growth of PCa with loss of AR activity may be driven by FGFRs signaling. Indeed, in an ARR2PBi^Pbsn−Cre^/TRAMP/fgfr1^loxP/loxP^ transgenic mouse model of PCa, tumors that escape *fgfr1* deletion or express *fgfr1* are poorly differentiated and metastatic phenotype, whereas those with *fgfr1* deletion are well-differentiated and phyllode phenotype [15]. Recently, Chiodelli et al. have reported that the combination therapy of pemigatinib, a FDA-approved FGFR inhibitor, with enzalutamide results in long-lasting tumor inhibition and prevention of CRPC relapse in TRAMP model of PCa [54]. All of these studies and our findings from present study suggest a determinant role of FGFR1 signaling in the mechanism of metastasis in advanced PCa and potential of anti-FGFR1 therapies such as anti-FGFR1 antibodies or inhibitors for treatment of advanced PCa after androgen deprivation therapy and AR-targeted therapy.

One has to acknowledge the limitations of this study. First, in addition to autocrine FGFs/FGFRs, other autocrine growth factor/their receptor loops such as VEGF/VEGFR2 and TGFα/EGFR may contribute the growth of DU145 cells [55], so that the contribution of these autocrine growth systems to the LDHA-aerobic glycolysis in this cell line or other prostate tumor cells remains further investigation. Second, DU145 cells are derived from metastatic prostate tumors in the brain and are AR-negative [24], so that they probably only represent a small portion of metastatic CRPC such as DNPC subtype. Third, the present study shows the autocrine FGFs/FGFR1 signaling facilitates cell proliferation, migration, and survival of DU145 cells via LDHA-aerobic glycolysis, but if FGFRs such as FGFR2 on tumor cells of CRPC in PCa patients can be activated by FGFs by paracrine and endocrine mechanisms and lead to the progression of PCa through the Warburg effect are not investigated. Finally, our data demonstrated the “association” of FGFR1 signaling with LDHA-aerobic glycolysis; however, how these two interact with each other remains further investigation. Experiments such as using CRISPR-Cas9 system to knockout FGFR1 gene in DU145 to generate a new experiment system—FGFR1 null DU145 cells are needed to confirm the link between FGFR1 and LDHA with tumor cell phenotype.

## 5. Conclusion

The plasticity of prostate tumors is induced by their progression and therapeutic treatment, which is often presented by the conversion of AR-positive to AR-negative tumors and the lineage switch from adenocarcinomas to NE phenotype in advanced PCa [56], and as a result, it confers resistance of the advanced PCa to AR-targeting and other therapies. Recent evidence indicates that metabolic adaptations such as aerobic glycolysis support growth and survival of prostate tumor cells beyond androgen signaling [57]; thus, targeting the signaling that regulates aerobic glycolysis could lead to develop novel therapies in the management of patients with CRPC. Our data further confirm that the aerobic glycolysis in AR-independent PCa DU145 cells can be regulated by autocrine FGFs-FGFR1 signaling via LDHA, and it mainly contributes to their proliferation, migration, and survival in cultures as well as tumorigenicity in a xenograft model, suggesting that targeting key metabolic control points of aerobic glycolysis, such as FGFRs or LDHA, might warrant investigation as potential drug target candidates for CRPC.

## Data Availability

The data from the experiments presented in this study are available from the corresponding authors upon request.
